# Structural Changes in the Cap of Rv0183/mtbMGL Modulate the Shape of the Binding Pocket

**DOI:** 10.3390/biom11091299

**Published:** 2021-09-01

**Authors:** Christoph Grininger, Mario Leypold, Philipp Aschauer, Tea Pavkov-Keller, Lina Riegler-Berket, Rolf Breinbauer, Monika Oberer

**Affiliations:** 1 Institute of Molecular Biosciences, University of Graz, 8010 Graz, Austria; Christoph.Grininger@uni-graz.at (C.G.); Philipp_Aschauer@hms.harvard.edu (P.A.); tea.pavkov@uni-graz.at (T.P.-K.); lina.riegler-berket@uni-graz.at (L.R.-B.); 2 Institute of Organic Chemistry, Graz University of Technology, 8010 Graz, Austria; mario.leypold@outlook.com (M.L.); breinbauer@tugraz.at (R.B.); 3 BioHealth Graz, 8010 Graz, Austria; 4 BioTechMed Graz, 8010 Graz, Austria

**Keywords:** *Mycobacterium tuberculosis*, Rv0183, lipase, covalent inhibitors, X-ray crystallography, conformational change, monoacylglycerol lipase

## Abstract

Tuberculosis continues to be a major threat to the human population. Global efforts to eradicate the disease are ongoing but are hampered by the increasing occurrence of multidrug-resistant strains of *Mycobacterium tuberculosis*. Therefore, the development of new treatment, and the exploration of new druggable targets and treatment strategies, are of high importance. Rv0183/mtbMGL, is a monoacylglycerol lipase of *M. tuberculosis* and it is involved in providing fatty acids and glycerol as building blocks and as an energy source. Since the lipase is expressed during the dormant and active phase of an infection, Rv0183/mtbMGL is an interesting target for inhibition. In this work, we determined the crystal structures of a surface-entropy reduced variant K74A Rv0183/mtbMGL in its free form and in complex with a substrate mimicking inhibitor. The two structures reveal conformational changes in the cap region that forms a major part of the substrate/inhibitor binding region. We present a completely closed conformation in the free form and semi-closed conformation in the ligand-bound form. These conformations differ from the previously published, completely open conformation of Rv0183/mtbMGL. Thus, this work demonstrates the high conformational plasticity of the cap from open to closed conformations and provides useful insights into changes in the substrate-binding pocket, the target of potential small-molecule inhibitors.

## 1. Introduction

Tuberculosis (TB) is one of the top causes of death from a communicable disease worldwide and a predominant cause of death from a single infectious agent, even during the ongoing COVID-19 pandemic. Despite a steady decline of case numbers over the last few decades, only a few countries are on track to achieve the goals set by the WHO and their “End TB” Strategy. Infections with the etiologic agent *Mycobacterium tuberculosis* (Mtb) and the occurrence of multidrug- and rifampicin-resistant strains (MDR/RR-TB) [1] will likely be further accelerated, even though the reported case numbers dropped significantly during 2020. The drop is likely attributed to the restriction of movement, personal economic impacts, and global reallocation of resources to the COVID-19 response. The first modeling attempts suggest a rise in the TB death rate in the upcoming years, which further increases the demand for new treatment strategies.

The life cycle of Mtb includes a dormant phase, where the bacterium enters a non-replicative state with downregulated metabolism. During this dormancy, the pathogen evades the host immune system and is insusceptible to common antibiotics [2]. Only a few enzymes of Mtb remain upregulated during the latent infection and present themselves as possible vulnerable targets for new treatment strategies. One of those enzymes is the mycobacterial monoacylglycerol lipase Rv0183, also termed mtbMGL [3], hydrolyzing monoacylglycerol (MG) into glycerol and an unesterified fatty acid (FA). Throughout the remainder of this manuscript, we will refer to the lipase as mtbMGL to emphasize its predominant catalytic activity. Mtb then uses FA in crucial metabolic pathways such as β-oxidation for energy homeostasis, polyketide lipid synthesis for cell membrane integrity [4], and storage in triacylglycerol (TG) containing intracellular lipid inclusions [5,6].

Interference with the lipid metabolism of Mtb is a promising strategy in the development and adaptation of treatment regimens for TB. The lipase inhibitors tetrahydrolipstatin (THL) and an oxadiazolone compound were found to inhibit several mycobacterial enzymes belonging to the hormone-sensitive lipase (HSL) family that severely affect the growth of Mtb and reduce the lipolysis of lipid bodies [7,8,9]. The expression levels of several lipase forms of Mtb have an impact on drug susceptibility and virulence. For example, elevated expression levels of the triacylglycerol lipase LipY leads to increased virulence [10]. LipF is reported to be a lipase and carboxylesterase capable of hydrolyzing the short-chain fatty acid TG. The overexpression of LipF in *Mycobacterium smegmatis* (Msm) leads to higher sensitivity of the bacterium to rifampicin (RIF) and streptomycin (STR) [11,12]. Overexpression of the esterase/lipase LipX of Mtb is linked to increased virulence and expression in Msm leads to a gain of stress tolerance, possibly due to an altered profile of cell-wall lipids [13]. The altered levels of LipG also show an effect on the cell wall lipid composition, and the disruption of the ortholog in Msm leads to an augmented tolerance to RIF and isoniazid (INH) [14].

Our work focuses on mtbMGL since the genetic disruption of the Msm ortholog leads to a drastic change in colony morphology, impaired growth on monoolein (MO)-containing media, and increased susceptibility to RIF [15]. This is also of special interest in context with the abovementioned retained activity of mtbMGL during the dormant phase of infection. It is also important to note that mtbMGL has been linked to the prodrug activation of new possible anti-tubercular compounds, although the exact mechanism and compartment of conversion of the prodrug is still under discussion [16]. These findings highlight the complexity of the interplay of lipolytic enzymes in Mtb and demand ongoing research. Understanding the structure of the involved enzymes is a decisive element in explaining their function.

The biochemical characterization of mtbMGL showed a strong preference for monoacylglycerols (MG) and suggests it as a secreted lipase [17]. mtbMGL belongs to the family of serine hydrolases, with an α/β-hydrolase core fold and a distinct cap domain. The first crystal structure was solved in our group and showed a remarkable similarity in its cap domain to that of the human homolog MGL (hMGL) [18]. To fully understand the activity of mtbMGL, it is still necessary to confirm the proposed substrate interactions [18] experimentally and to study conformational changes of the cap, both of which are addressed in this work. Similar cap domains are also found in MGLs from other organisms (human [19], yeast [20], bacteria [21], archaea [22]) and are thought to be a distinctive feature [23]. For hMGL, extensive research was performed on the connection between activity and structural changes in the cap domain. The interaction of hMGL with a membrane phospholipid bilayer leads to an open cap conformation and enhances the enzyme’s activity [24]. Moreover, single point mutations that are far from the cap domain infer changes in the conformational equilibrium between the open and closed forms of the enzyme [25].

In this work, we present two crystal structures of an mtbMGL surface-entropy reduction (SER) variant at high resolution. One structure shows a complex with a substrate analog, mimicking the tetrahedral transition state in the active site and ligand interactions with the cap domain. The second crystal structure displays a closed cap conformation and illustrates cap plasticity and the specific cap-closing mechanism of mtbMGL. This knowledge will improve the rational search for inhibitors of this enzyme in the attempt to propose new treatment strategies in the fight against TB.

## 2. Materials and Methods

### 2.1. Cloning, Expression and Purification

Sites for surface entropy reduction (SER) mutations were predicted with the online server SERp of UCLA [26]. The plasmids with the proposed mutations were prepared by site-directed mutagenesis, using the Q5 site-directed mutagenesis kit (NEB, Cat. # E0554S). The template plasmid was described in a previous publication and contains the codon-optimized gene for mtbMGL in the pProExHtb vector [18]. The following primers were used:

K74A forward primer 5′-TAGCGGCGGTGCACGGGTCTTG-3′,
K74Areverse primer 5′-CGCCCATGACCACGGTGA-3′,
Q164A forward primer 5′-TTTACCGGTAGCGGAACTCGATTT-3′,
Q164A reverse primer 5′-AAATCGAGTTCCGCTACCGGTAAA-3′,
E165A forward primer 5′-CCGGTACAGGCACTCGATTTC-3′,
E165A reverse primer 5′-GAAATCGAGTGCCTGTACCGG-3′,
K249A forward primer 5′-GTACAGCTGGCGGAGTATCCG-3′,
K249A reverse primer 5′-CGGATACTCCGCCAGCTGTAC-3′.

The resulting plasmids were sequenced for verification of the mutations (Microsynth Austria GmbH (Vienna, Austria) and were further used for expression and purification according to previously established methods [18]. Briefly, the expression was performed in *Escherichia coli* BL21 (DE3) Codon Plus in LB broth, at 18 °C overnight. The purification was achieved with Ni affinity chromatography (HisTrap Fast Flow 5 mL column, Cytiva, Uppsala, Sweden) and size exclusion chromatography (Superdex 200 16/60, Cytiva, Uppsala, Sweden) in 50 mM Tris-HCl, 150 mM NaCl. Monomeric protein was concentrated to 10 mg/mL with centrifugal filters (Amicon Ultra, 10 kDa molecular weight cut-off, Merck Millipore, Darmstadt, Germany) and flash-frozen in 50 µL aliquots in liquid nitrogen, for storage at −80 °C.

### 2.2. Crystallization, Data Processing and Refinement

Crystallization experiments were performed with an ORYX 8 robot from Douglas Instruments (Hungerford, UK). The screening was performed using commercial screens from Molecular Dimensions in 96-well plates for sitting-drop vapor-diffusion experiments. All crystallization trials were carried out in protein crystallization cabinets (RuMED, Laatzen, Germany) at 20 °C. To prevent aggregation in the crystallization setup, decreased protein concentration and the addition of L-Arg/L-Glu and L-Pro were tested [27,28]. Proline showed beneficial effects and was subsequently added to the protein solution prior to the crystallization setup (50 mM final concentration). For the setup, 0.5 µL of protein solution (10 mg/mL) was mixed with 0.5 µL reservoir solution. First, protein crystals for the variant mtbMGL K74A were obtained in condition 46 of Wizard Classic 4 (30% 2-propanol, 30% PEG 3350, 100 mM Tris-HCl pH 8.5). After a 2D-optimization of the PEG 3350 and 2-propanol concentrations, the obtained crystal clusters from one condition (10% 2-propanol, 33% PEG 3350, and 100 mM Tris-HCl pH 8.5) were used for the preparation of a seeding stock (1 µL of crushed crystal solution, added to 50 µL of the original condition). Further optimizations were performed by mixing 0.5 µL of protein solution (10 mg/mL) with 0.5 µL screen condition and 0.2 µL of 1/1000 diluted seed stock. The final condition for the closed cap structure was 15% 2-propanol, 24% PEG 3350, and 100 mM Tris-HCl, pH 8.5. For co-crystallization of mtbMGL K74A with the substrate analog Hexadecyl butylphosphonofluoridate (referred to as Maglipan throughout the text), the concentrated protein solution was diluted with crystallization buffer (50 mM Tris-HCl pH 8.5, 150 mM NaCl, 50 mM proline) to approximately 1 mg/mL and incubated with the substrate analog (dissolved in DMSO) at a molar ratio of 1:10 (protein:substrate analog), not exceeding a DMSO concentration of 1%. After incubation for 1 h at room temperature, the protein solution was again concentrated in a centrifugal filter, washed with crystallization buffer and again concentrated to 10 mg/mL. Sitting-drop vapor-diffusion experiments were performed again in 96-well plates, and 0.5 µL protein solution (10 mg/mL) was mixed with 0.5 µL of the reservoir solution and 0.2 µL of the previously described seeding stock (1/1000 dilution). Initial crystals were observed in condition D10 of the screen JCSG+ (0.2 M calcium acetate, 40% PEG 300, 100 mM sodium cacodylate pH 6.5). After extensive optimization, well-diffracting crystals were obtained in a condition containing 30% PEG 300, 125 mM calcium acetate and 100 mM Tris-HCl pH 8.5.

Diffraction data for the crystal with the closed-cap structure were measured at the EMBL beamline P13 (Hamburg, Germany) [29]. The data were indexed and integrated with XDS (BUILT = 20210205) [30]. The data of the co-structure crystal were measured at the DESY beamline P11 (Hamburg, Germany) [31], and indexed and integrated with DIALS (version 1.14.13) [32]. The structures were solved by molecular replacement via Phaser [33], using chain C from the wild-type (wt) mtbMGL structure (PDB-code 6EIC [34]) as a search template. The initial models were completed manually in Coot (version 0.9.5) [35] and refined with Phenix (version 1.19.2-4158) [36]. The restraints for the novel ligand Maglipan were calculated with eLBOW [37]. The final structure was evaluated using MolProbity(version 4.5.1) [38]. Detailed data processing and structure refinement statistics are summarized in Table 1. All structure-related pictures were generated with PyMOL (The PyMOL Molecular Graphics System 2.4.1, Schrödinger, New York, NY, USA). The atomic coordinates and structure factors have been deposited to the Protein Data Bank with the accession codes 7OZM (closed-cap conformation) and 7P0Y (substrate analog complex structure).

### 2.3. MGH Activity Assay

For measuring the activity of the mtbMGL variants, 50 µL of substrate solution (1 mM 1-oleoyl-rac-glycerol (Merck, Darmstadt, Germany) solubilized in 55 mM phosphate buffer pH 7.3 and 5 mM CHAPS) and 100 µL free glycerol reagent (solubilized in 55 mM phosphate buffer, Sigma-Aldrich) were used as reaction mix. After adding 5 µL of 30 nM protein and 20 min incubation at 37 °C, the reaction was stopped with 15 µL of 4 M Na acetate pH 4.5, and the developed color was read out at 560 nm. The detailed assay setup and substrate preparation procedure have been described in previous publications [18,21].

### 2.4. Synthesis of Maglipan

Hexadecyl butylphosphonofluoridate (“Maglipan”) was synthesized as a substrate-mimicking inhibitor. All commercially available reagents and solvents were purchased from Sigma-Aldrich, Alfa Aesar, ABCR, Fisher Scientific, Acros Organics, Roth or VWR, and were used without further purification except when otherwise stated. All reactions were carried out under an argon atmosphere using established Schlenk techniques. For the synthesis, an oven-dried, evacuated and argon-purged 80 mL Schlenk flask, equipped with a Teflon-coated magnetic stirring bar, was charged with 1-butylphosphonic acid (331 mg, 2.40 mmol, 1.20 eq). It was dissolved in 20 mL of anhydrous 1,2-DCE:DMF = 9:1 (*v*/*v*) under an argon atmosphere, and 1-hexadecanol (485 mg, 2.00 eq, 1.00 eq) as well as 4-(dimethylamino)pyridine (4-DMAP, 12.2 mg, 100 µmol, 5.0 mol%) were added to the colorless solution, respectively. The resulting colorless solution was cooled in an ice bath to 0 °C. In an argon counterflow, N-(3-dimethylaminopropyl)-N’-ethylcarbodiimide hydrochloride (EDC.HCl, 575 mg, 3.00 mmol, 1.50 eq) was added in one portion; immediately afterward, the ice bath was removed, and the colorless suspension was stirred at room temperature under argon for 10 min. Subsequently, the colorless suspension was heated in an oil bath to 80 °C for 24 h. The colorless solution was cooled to room temperature and the solvent was carefully removed in the vacuum of an oil pump. For hydrolysis of the formed pyrophosphonates, the bright yellow, viscous residue was allowed to stand at room temperature in an open reaction vial for 72 h. Afterward, the residue was dissolved in 100 mL EtOAc, and the organic solution was washed with half-saturated NH_4_Cl (2 × 50 mL). The organic layer was dried over MgSO_4_, was filtered, and the solvent was removed on a rotary evaporator. Finally, the light yellow, viscous crude material was purified via flash column chromatography (80 g SiO_2_, column: 30.0 × 3.0 cm, eluent: 300 mL cyclohexane:EtOAc = 1:1 (*v*/*v*), then EtOAc:MeOH = 3:2 (*v*/*v*) + 0.01% AcOH, Rf = 0.47) to yield monohexadecyl butylphosphonate (638 mg, 1.76 mmol, 88%) as a colorless liquid.

An oven-dried, evacuated and argon-purged 100 mL Schlenk flask was charged with monohexadecyl butylphosphonate (544 mg, 1.50 mmol, 1.00 eq). It was suspended in 25 mL anhydrous DCM, and the colorless suspension was cooled in an ice bath to 0 °C under an argon atmosphere. (Diethylamino)sulfur trifluoride (DAST, 297 µL, 363 mg, 2.25 mmol, 1.50 eq) was added, and the resulting colorless solution was stirred in the ice bath under argon for an additional 2 h. The colorless solution was diluted with 25 mL cold DCM, and the organic layer was washed with cold H_2_O (2 × 25 mL) within 5 min. The colorless organic layer was dried over MgSO_4_, and was filtered into an oven-dried, evacuated and argon-purged 20 mL Schlenk flask. The solvent was carefully removed in the vacuum of an oil pump to yield Maglipan (541 mg, 1.48 mmol, 99%) as a colorless liquid. The phosphonofluoridate could be stored in the Schlenk flask under an argon atmosphere in a freezer at −24 °C for several months without significant degradation. For analytical purposes of the compounds, ^1^H-, ^13^C-, ^31^P- and ^19^F-NMR spectra were recorded on a Bruker AVANCE III 300 spectrometer (^1^H: 300.36 MHz; ^13^C: 75.53 MHz) or Varian Unity Inova 500 (^1^H: 499.88 MHz; ^13^C: 125.69 MHz, ^31^P: 202.35 MHz, ^19^F: 470.35 MHz). Chemical shifts were referenced to the residual proton and carbon signal of the deuterated solvent, respectively (CDCl_3_: δ = 7.26 ppm (^1^H), 77.16 ppm (^13^C)). Signal multiplicities were abbreviated as s (singlet), d (doublet), dd (doublet of doublet), t (triplet), q (quadruplet), p (pentet) and m (multiplet). Additionally, quaternary carbon atoms were designated as C_q_. Deuterated solvents for nuclear resonance spectroscopy were purchased from Euriso-Top (Saint-Aubin, France). Analytical thin-layer chromatography (TLC) was carried out on Merck TLC silica gel 60 F254 aluminum sheets, and spots were visualized by UV light (λ = 254 and/or 366 nm) or by staining with cerium ammonium molybdate (CAM), followed by the development of the stains in the heat.

^1^H-NMR (300.36 MHz, CDCl_3_, 25 °C): δ = 4.22–4.11 (m, 2H), 1.98–1.77 (m, 2H), 1.75–1.55 (m, 4H), 1.49–1.17 (m, 30H), 0.95–0.84 (m, 6H).

^13^C-NMR (75.53 MHz, CDCl_3_, 25 °C): δ = δ 67.09 (d, *J* = 7.4 Hz), 32.05 (s), 30.50 (d, *J* = 5.7 Hz), 29.81 (s, 3 × C), 29.79 (s, 2 × C), 29.75 (s), 29.66 (s), 29.59 (s), 29.49 (s), 29.19 (s), 25.47 (s), 24.12 (dd, *J* = 143.5, 22.6 Hz), 24.08 (d, *J* = 5.6 Hz), 23.55 (d, *J* = 17.2 Hz), 22.81 (s), 14.22 (s), 13.53 (s).

^31^P-NMR (202.35 MHz, CDCl_3_, 25 °C): δ = 31.81 (d, *J* = 1069 Hz).

^19^F-NMR (470.35 MHz, CDCl_3_, 25 °C): δ = –65.04 (d, *J* = 1069 Hz).

## 3. Results

### 3.1. Surface Entropy Reduction (SER) Variants Remain Catalytically Active

In previous work, our group demonstrated that mtbMGL can be expressed and purified in a native and catalytically active form. The crystallization of wt mtbMGL was possible, but the reproducibility of crystals was limited. Previous structures of bacterial MGL had shown that “snapshots” of different existing conformations can be captured when analyzing simply a higher number of structures in the presence or absence of ligands [21,39]. To improve the crystallization likelihood of mtbMGL and therefore increase the chances for capturing different conformational states, SER variants were generated. The SER approach draws on the observation that surface-exposed residues with high conformational entropy (especially lysines, glutamines, and glutamates) are less likely to participate in protein–protein interactions and thus often impede or slow down crystallization. To improve the chances for proteins to assemble in a crystalline lattice, clusters of such high-entropy residues are interrupted with low-entropy residues, such as alanines [40]. The SER-prediction server suggested 3 clusters for mutagenesis, namely, K249A and E250A in Cluster 1, Q164A and E165A in Cluster 2, and K74A in Cluster 3. We selected four mutation sites individually for each variant, based on our previous structure of mtbMGL, especially considering the high amount of surface exposure and the least likelihood of impacting enzymatic activity.

**Figure 1:**
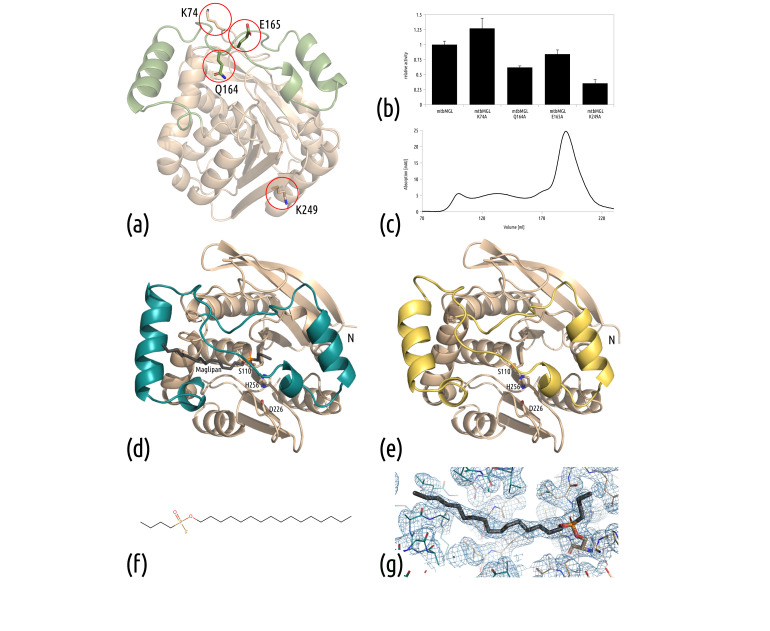
(**a**) Positions of the SER mutations displayed on the wt mtbMGL structure (PDB: 6EIC), cap domain in green, core fold in beige. (**b**) Monoacylglycerol hydrolase activity of mtbMGL K74A, mtbMGL Q164A, E164A and K249A, relative to wt mtbMGL. Here, 100% of wt mtbMGL activity corresponds to 276 ± 16 μmol glycerol/(h*mg protein). (**c**) Size exclusion chromatogram of mtbMGL K74A after Ni-affinity purification. The monomeric fraction (eluting around 200 mL) was used for crystallization. (**d**) The complex structure of the SER variant mtbMGL K74A with the covalently bound substrate analog Maglipan. The catalytic triad is labeled and shown as a stick representation. (**e**) The overall structure of the surface entropy variant mtbMGL K74A with a closed cap conformation. The catalytic triad is labeled and shown in sticks representation. (**f**) The unbound structure of the substrate analog Maglipan (**g**) Maglipan from chain A of the complex structure 7P0Y, bound to S110 with electron density (2F_o_–F_c_ at a 1.0 sigma contour level). The surrounding protein residues are shown as line representations.

The positions of the altered residues are all located at the surface of the protein. Q164 and E165 are located within the cap domain, whereas K74 and K249 are positioned on the surface of the α/β-hydrolase core fold. K74, Q164 and E165 are found in loop regions, whereas K249 is located in β-strand 8 of the core fold (Figure 1a). The variants K74A, Q164A, E165A and K249A were successfully generated by site-directed mutagenesis, then expressed and purified. All variants were tested for their ability to hydrolyze monoacylglycerol; they retained catalytic activity, although the variants mtbMGL K74A and mtbMGL E165A exhibited hydrolytic activity closest to the wt mtbMGL (Figure 1b). Similar to the wt enzyme, the SER variants are mainly purified as monomers (determined by SEC, Figure 1c). All variants were used for crystallization trials and resulted in heavy precipitation. In a first attempt, the protein concentration was reduced. The addition of a 50 mM equimolar mixture of L-Glu/L-Arg or 50 mM L-Pro to the crystallization setup was tested. Proline was seen to reduce precipitation and was used in further crystallization attempts. Only mtbMGL K74A yielded well-diffracting crystals.

### 3.2. New Structures Reveal a Closed-Cap Conformation and Show the Bound Substrate Analog Maglipan

The protein crystallized in different conditions with and without the covalently bound substrate analog Maglipan (Figure 1d–g). This new inhibitor (Figure 1f) resembles the substrate closely and lacks the azido-moiety in the head group that was present in previously used substrate analogs [21].

The crystals of the mtbMGL K74A without a ligand diffracted to a resolution of 2.15 Å (PDB: 7OZM), with the substrate analog they diffracted to a resolution of 2.25 Å (PDB: 7P0Y). The structures were solved by molecular replacement with the structure of wt mtbMGL (PDB: 6EIC [34]). The closed-cap structure was refined to R values of R_free_ = 24.9% and R_work_ = 19.5%, while the substrate analog complex-structure was refined to R_free_ = 29.4% and R_work_ = 25.3%. Overall, the refinement statistics for the complex structure are slightly worse compared to structures of similar resolution. This can be explained by the missing stabilizing crystal contacts for chain B; therefore, there is high flexibility in this chain. The loop regions of chain B are poorly defined, and the B-factors are higher than in chain A; the average B-factor for chain A is 35.9 Å^2^ and for chain B, 56.0 Å^2^. Nevertheless, there is density visible for the ligand in both chains (Figure 1g). The detailed statistics of data processing, merging and refinement are shown in Table 1.

**Figure 2:**
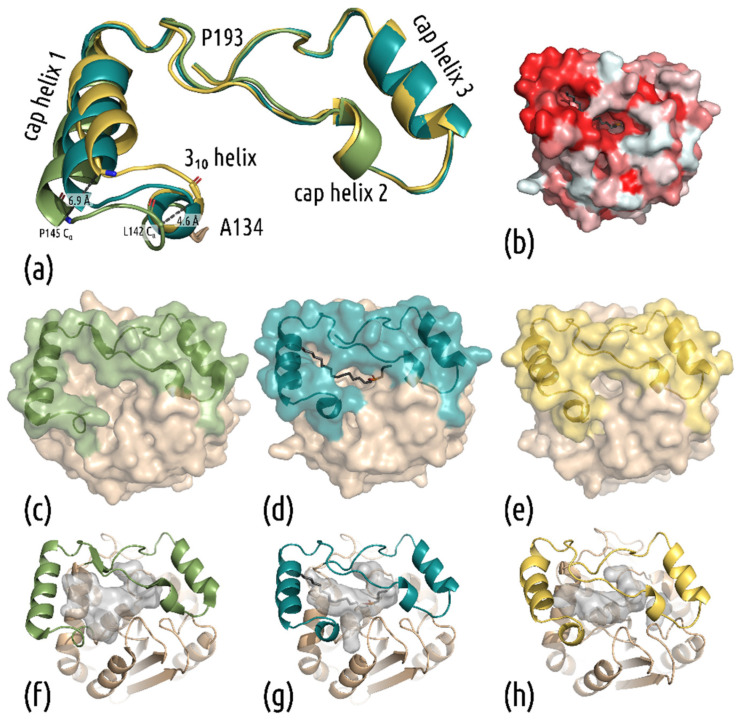
(**a**) Overlay of the three cap conformations from wt mtbMGL (green), the complex structure with Maglipan and mtbMGL K74A (teal), closed-cap conformation (yellow). (**b**) The complex structure of mtbMGL K74A with Maglipan in surface representation, colored by hydrophobicity (white hydrophilic, red hydrophobic, according to the Eisenberg hydrophobicity scale [41]. Surface representations of (**c**) wt mtbMGL, (**d**) complex structure, (**e**) closed-cap structure. Cavities are shown for (**f**) wt mtbMGL, (**g**) complex structure, (**h**) closed-cap structure. The cap domain is shown in different colors, the α/β hydrolase core fold in beige.

Overall, the two new structures are highly similar to that of wt mtbMGL (PDB: 6EIC [34]) with an overall rmsd of 0.346 Å (241 residues, 6EIC_B to 7OZM) and 0.264 Å (235 residues, 6EIC_B to 7P0Y_A). mtbMGL K74A harbors an α/β-hydrolase fold formed by 8 β-strands with a total of 6 α-helices on both sides in the so-called core domain (residues Thr2-Val137 and Ala194-Leu279). A cap region (defined as residue Ala138-Pro193, Figure 2a), comprising predominantly helices and loop regions, resides on top of the core domain. No significant changes in the α/β-hydrolase core were observed. The active site is formed by residues Ser110, His256 and Asp226 (Figure 1d,e). The oxyanion hole in mtbMGL K74A, the pocket that stabilizes the negatively charged transition state through hydrogen bonding, is formed by the backbone amide nitrogens of the residues Leu39 and Met111. In the open conformation, observed in wt mtbMGL, access to the active site via a primarily hydrophobic entrance pocket is given by a wide opening of the cap helix 1 and the loop residues Gly160–Gln164 (Figure 2a–c). Parts of this hydrophobic binding pocket are still visible in the complex structure of mtbMGL K74A, with Maglipan displaying an intermediate conformation between the open and closed forms (Figure 2b).

Interestingly, the cap domain covering the active site of the enzyme shows conformational changes when comparing the three crystal structures among each other (Figure 2). Without the substrate analog, mtbMGL K74A crystallized in a completely closed cap conformation. It can be seen in Figure 2 that the first helix of the cap domain (cap helix 1, Figure 2a) distorts and rolls over the entrance tunnel (Figure 2e). In the complex structure, cap helix 1 adopts an intermediate stage between the open and closed conformation. The ligand dives deeply into the lipase to the active site within the core domain, whereas the end of the alkyl chain still projects into the exterior of the substrate-binding cavity. We observed electron density throughout the entire substrate entrance tunnel of both chains, up to the active site Ser110, into which we could nicely fit the substrate analog Maglipan (Figure 1g).

In comparison to the open-cap conformation of mtbMGL, the substrate-bound structure also shows changes in the cap domain. At the N-terminal end of the cap domain, the loop leading to the first helix forms a single turn of a 3_10_-helix in both structures of mtbMGL K74A. This change forces the N-terminal part of cap helix 1 toward the active site. In the closed-cap conformation, this leads to the contacts of cap helix 1 with the consecutive loop region (Gly160–Gln164). In the complex structure, the ligand is positioned between the mentioned parts of the cap domain. The comparison of the different structures is shown in Figure 2. The aligned cap domains depict the rolling distortion of the helix (Figure 2a). Due to this movement, a substrate can either have full access to enter the tunnel leading to the active site, be completely blocked from entering, or be held in place (Figure 2c–e). The cavity leading to the catalytically active serine changes its shape from a broad tunnel in the open-cap conformation to a tight sleeve around the substrate, to a cavity that is nearly completely separated from the exterior (Figure 2f–h).

### 3.3. Interactions in the Active Site

**Figure 3:**
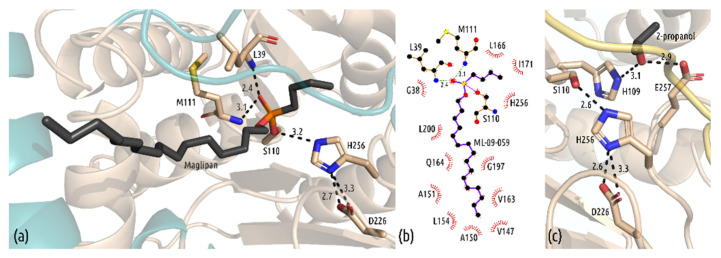
(**a**) Close-up view of the covalently bound substrate analog Maglipan, with interactions in the active site. The catalytic triad (S110, H256, D226) and the residues forming the oxyanion hole (M111, L39) are shown in stick representation. (**b**) Interactions of Maglipan with mtbMGL K74A calculated with Ligplot+. (**c**) 2-Propanol, found in the active site of mtbMGL K74 with closed-cap conformation. Interacting residues (H109, E257) are shown in stick representation; the catalytic triad is shown for orientation.

Our structure reveals detailed insight into the interactions of the ligand with the binding site (Figure 3). The central phosphorus atom of the ligand bound covalently to Ser110, and the extended aliphatic chain expands through the hydrophobic tunnel formed by residues Val147, Ala150, Ala151, Leu154, Val163, Gln164, Gly197 and Leu200. Cap helix 1 (Pro145-Val157) and the residues from the subsequent loop (Val163, Gln164) interact with the tip of the alkyl chain (Figure 3b). The complex structure of mtbMGL K74A, with the bound substrate analog Maglipan, mimics the transition state intermediate of 1-oleoyl-rac-glycerol (1-OG) in the active site of the enzyme. The active site of serine, Ser110, and the central phosphorus atom form a covalent bond; fluorine has been replaced in the nucleophilic attack of the hydroxy group of Ser110. The backward-facing oxygen atom O19 of the covalently bound compound Maglipan is also coordinated by two hydrogen bonds from the backbone of Leu39 and Met111 that form the oxyanion hole in the active site (Figure 3a). The short butyl group of the substrate analog faces toward the end of the long cavity of the active site. Previous docking experiments predicted that the glycerol moiety would form hydrogen bonds with Tyr181 and Glu257 during the reaction of mtbMGL with 1-OG [34]. Since the ligand Maglipan lacks any hydroxy groups, this interaction cannot be confirmed. The long aliphatic chain of the ligand interacts with various hydrophobic residues throughout the entrance tunnel, built from the α/β-hydrolase core fold and the cap domain. The end of the chain (C1–C8) is positioned between the residues of the first cap helix (Val147, Ala150, Ala151, Leu154) and the adjacent loop region (Val163) (Figure 3b). In the structure of mtbMGL K74A with a closed-cap conformation, the additional electron density in the active site cavity was explained by 2-propanol, originating from the crystallization condition. The hydroxy group is in hydrogen bonding distance to Glu257 and His109 (Figure 3c).

## 4. Discussion

The structure of wildtype mtbMGL displayed an open-cap conformation, and crystallization was successful only after multiple rounds of optimization [18]. To improve the success rate of our crystallization trials, we introduced SER mutations on the gene of mtbMGL. The selected amino acid exchanges had only a minor impact on the activity of the enzyme, yet we identified new crystallization conditions with the variant mtbMGL K74A. The structures of mtbMGL K74A presented herein exhibit high overall similarity to the previously published wt mtbMGL structure, apart from significant differences in the cap domain of the enzyme. This domain forms a Z-like shape and is formed by two α-helices, a helical turn and a short β-strand. Native mtbMGL K74A crystallized in a completely closed-cap conformation. The first α-helix between the residues P145 and V157 distorts and closes the entrance to the cavity leading to the active site by a distortion of the helix and a large movement of the N-terminal part towards the adjacent loop region. Following this rolling movement, the N-terminal loop of the cap domain, connecting β-strand 6 of the α/β-hydrolase core fold with the first helix in the cap domain, forms a single turn of a 3_10_ helix. Despite the low sequence homology of MGLs, they share the common α/β-hydrolase core fold and the conserved cap architecture [23]. Extensive research was performed on the relationship between conformational changes in the cap domain of hMGLs, and their activities in relation to open and closed states [19,22,24,25,42]. Combined with the changes presented herein in the cap domain of mtbMGL, new questions arise. Is the closed-cap conformation promoted by the exchange of Lys74 to alanine? A permanently closed cap is highly unlikely since mtbMGL K74A is still catalytically active and crystallized in complex with the substrate analog Maglipan. To enable access of the ligands 1-OG or Maglipan to the binding pocket, at least a temporary opening of the cap is necessary. It is assumed that the fatty acids as final products exit the catalytic binding pocket on the same path by which the substrate MG has entered. Smaller access paths to the catalytic site were observed in the cap region for human MGL in apo form as well as the complexed form (proximity of residues Ile179, Tyr194, Arg202) and MGL from *Bacillus* sp. H257 (bMGL) (proximity of residues Ile145, G146, S145, Glu160, Glu156) [19,39,43,44]. These tunnels could represent exit holes to glycerol as the first reaction product, but experimental studies that clearly prove that these are dedicated “glycerol exit holes” are still pending. In the closed or complexed structure of mtbMGL K74A, this hole is closed or very narrow, due to an inward orientation of L166, which would correspond to the gating residue of Ile145 in bMGL or Ile178 in human MGL. The closed ligand-bound form of human MGL reported by Schalk-Hihi et al. also displays a large rolling motion of cap-helix 1 (termed α-helix 4 in that publication) [42]. This potential glycerol exit hole is also completely closed off by small sequential conformational changes in the loop regions connecting cap helix 1 with cap helix 2, and a pronounced inward orientation of Ile179 [42].

In addition to the closed cap conformation, the complex structure of mtbMGL K74A with the substrate analog Maglipan was solved. So far, the structural knowledge of substrate binding of mtbMGL was based on docking experiments with the wild-type structure [18]. In this work, we can confirm the results of these docking experiments. The tetrahedral transition state is stabilized by the oxyanion hole comprising the backbone nitrogens of the residues Leu39 and Met111. The aliphatic chain of the ligand is held in place by various hydrophobic interactions, with residues in the core fold and the cap domain. Comparing the structure of the cap domains of all three available mtbMGL structures, it is evident that for the substrate binding, the flexibility of the first cap helix is also necessary.

With the new structures, the cavity around the active site of the enzyme, as well as the changes of the cavity as result of the changes in the cap, can be described in more detail. This is the next step in rationalizing the drug design for this enzyme. mtbMGL is a protein expressed during the whole life cycle of *M. tuberculosis,* including dormancy [3]. Inhibition of this lipase or other active enzymes in the dormant phase could open up a new opportunity in TB treatment. Newly developed compounds or modified drugs sometimes require enzymatic activation from the administered prodrug to the active compound [16]. Obviously, the generation of inhibitor-induced resistance mechanisms against otherwise active drugs has to be avoided. Therefore, inhibitors of mtbMGL—like those of any other enzymes in *M. tuberculosis*—have to be administered with great caution when combined with different compounds. This newly gained knowledge can be used for the rational design of inhibitors fitting into the open cap conformation, but also into the intermediate state and the closed cap conformation. Additionally, any structure-based drug development effort will benefit from including data on conformational changes in the binding pocket. The different conformations in the cap region of experimentally derived crystal structures of MGLs from different organisms [18,19,20,21,22,43,44], elegant NMR, and hydrogen–deuterium exchange mass spectrometry studies on human MGL [24,25] or molecular dynamics approaches [22] should be taken into account in structure-guided approaches for compound screening [45]. These benefits can be envisaged for in silico docking approaches for repurposing drugs or in the design of novel, specifically binding inhibitors of proteins from numerous pathogenic organisms [46].

## Figures and Tables

**Table 1:** Data collection and refinement statistics.

	7OZM						7P0Y					
Data Collection												
Wavelength (Å)	1.072						1.0332					
Resolution range (Å)	39.77–2.15 (2.23–2.15)						40.78–2.25 (2.33–2.25)					
Space group	P 2 21 21						P 21 2 21					
Unit cell	a	b	c	α	β	γ	a	b	c	α	β	γ
(Å, °)	40.50	82.24	90.86	90	90	90	74.69	82.60	93.78	90	90	90
Total reflections	54,614 (5426)						145,938 (14,206)					
Unique reflections	16,663 (1655)						28,192 (2766)					
Multiplicity	3.3 (3.3)						5.2 (5.1)					
Completeness (%)	97.22 (97.93)						99.65 (99.50)					
Mean I/sigma(I)	7.27 (1.43)						20.99 (1.19)					
Wilson B-factor	29.19						36.18					
R-merge	0.1415 (0.7517)						0.149 (0.7195)					
R-meas	0.1669 (0.8893)						0.1654 (0.8019)					
R-pim	0.08599 (0.4619)						0.07091 (0.3494)					
CC1/2	0.991 (0.578)						0.99 (0.77)					
CC*	0.998 (0.856)						0.998 (0.933)					
Refinement												
No. of reflections	16,658 (1665 for test set)						28,118 (3571 for test set)					
R_work_/R_free_	0.1950/0.2500						0.2534/0.2951					
Non-solvent atoms	2126						4240					
Solvent atoms	133						141					
RMS (bonds, Å)	0.007						0.003					
RMS (angles, °)	0.86						0.61					
Ramachandran- favored (%)	97.1						97.05					
Ramachandran- allowed (%)	2.54						2.95					
Ramachandran outliers (%)	0.36						0					
Rotamer outliers (%)	0.45						0.92					
Clashscore	3.53						8.18					
Average B-factor (Å^2^)	30.0						45.6					

The highest resolution shell statistics are shown in parentheses.

## Data Availability

Data presented in this study are openly available in the Protein Data Bank (PDB) PDB ID: 7OZM and PDB ID 7P0Y.
